# HEAR US: a qualitative study of racial discrimination in Boston’s Chinatown and empowering change from within the community

**DOI:** 10.3389/fpubh.2023.1212141

**Published:** 2023-09-04

**Authors:** Siyu Chen, Yajing Luo, Kimberly R. Dong, Yoyo Yau, Ben Hires, Shiwei Liang, Alice M. Tang

**Affiliations:** ^1^Department of Public Health and Community Medicine, Tufts University School of Medicine, Boston, MA, United States; ^2^Friedman School of Nutrition Science and Policy, Tufts University, Boston, MA, United States; ^3^Boston Chinatown Neighborhood Center (BCNC), Boston, MA, United States

**Keywords:** Asian, Chinese, racial discrimination, stigma, mental health, COVID-19, qualitative study

## Abstract

**Objective:**

To qualitatively explore the impact of anti-Asian racism in a Chinese community in the greater Boston area.

**Methods:**

Individual semi-structured interviews (n = 27) were conducted between June and September 2021. Eligible participants were ethnic Chinese immigrants living in the Boston area, who were recruited through a community-based organization and by word-of-mouth. Interviews were conducted in Mandarin and Cantonese and translated into English. Data were coded and analyzed using a directed approach to content analysis.

**Results:**

The majority of participants reported personal experiences of anti-Asian racism, ranging from microaggressions to violent attacks. Although lockdown and isolation during COVID-19 affected all communities, the Chinese community suffered unique and prolonged trauma stemming from the fear of violent attacks against Asians. The older person/people, in particular, were severely isolated due to fear of exposure to anti-Asian hate crimes. Participants reported a variety of emotional, mental, and physical health effects associated with feelings of fear, anxiety, isolation, and powerlessness. Many preferred to engage in self-protective behavior changes rather than relying on external resources.

**Conclusion:**

Participants advocated for more education, community, and governmental support, and increased allyship between communities of color. These findings provide cultural context on the trauma this population faces and can inform further actions to address the wide range of reported health effects.

## Introduction

The COVID-19 pandemic placed a stark spotlight on social and racial disparities in the U.S., with Black, Indigenous, and People of Color (BIPOC) communities suffering disproportionately from the health and economic effects of the disease. People of Asian descent faced unique challenges and barriers during this time, stemming from the rise in racist rhetoric sparked by the reporting of initial cases of COVID-19 in Wuhan, China. However, bigotry toward Asian Americans is not a new phenomenon. Since Asians first arrived in the United States during the late 19th century, they have faced racial discrimination that has persisted over time. The oppressive policies aimed at Asian immigrants, coupled with conflicts between the Western and Eastern worlds in the 20th century, have deepened the animosity toward this community in the U.S. (1). The use of racist rhetoric to assign blame to specific ethnic groups for disease outbreaks is also not without precedence. Just as the Bubonic plague in the 19th century and the 2003 SARS outbreak triggered xenophobia, the advent of COVID-19 once again thrust Asian Americans into the spotlight due to widespread scapegoating (2, 3). This unfair targeting of Asian Americans can be attributed to the enduring stereotype that portrays them as “perpetual foreigners,” regardless of nationality or immigration status (4–6).

Since March 2020, tens of thousands of racist incidents against Asian Americans and Pacific Islanders (AAPIs) across the U.S. have been reported to *Stop AAPI Hate*, an online reporting center established by a group of AAPI civil rights organizations (7). A large proportion (70%) of the reported cases were verbal attacks including racial slurs, name-calling, and profanities, while a smaller proportion (9%) of them were physical attacks (7). Several nationwide surveys have been conducted during the past three years revealing the oppressive and stressful environment faced by members of Asian communities. Statistics from a collection of survey data demonstrated increases in hate incidents targeting Asian American and Native Hawaiian/Pacific Islanders (AANHPIs) in 2020 and 2021 (8). Nearly 30% of AANHPI participants from the California Health Interview Survey reported having witnessed unfair treatment toward another AANHPI person, while 75% of Asian participants in the AA & NHPI COVID-19 Needs Assessment Project believed that the living environment in the US had become more threatening for their race (8, 9). The National Asian Pacific American Women’s Forum (NAPAWF) survey revealed that more than two-thirds of AAPI women experienced racism or discrimination in 2020, with East Asian women reporting a much higher propensity to feel insecure compared to other Asian ethnic groups (51% vs. 33%) (10). Results from the Understanding America Study showed that Asians were more likely to perceive COVID-19-associated discrimination compared to non-Hispanic Whites (adjusted odds ratio = 3.2), and that perceived discrimination was related to a 30% increase in mental distress points based on the 4-item Patient Health Questionnaire score (11). Furthermore, an analysis of the 2020 Healthy Minds Study (HMS) found that over 60% of AAPI student respondents who experienced COVID-related racial discrimination met the criteria for at least one mental health condition (12).

The plethora of articles on the resurgence of anti-Asian racism in the U.S. following the pandemic has primarily been quantitative in nature (7–18). To date, only two qualitative studies have been published on this topic – one in Asian healthcare workers in Canada and the other in international students (16, 19). No studies have documented the lived experiences of a largely immigrant population of Asians in a community setting. The purpose of this study is to provide richer context to the numbers and types of experiences reported in quantitative surveys. Using in-depth qualitative interviews, this study further explores the thoughts, emotions, and reactions around personal experiences of anti-Asian hate, explores access and availability of local community support and resources, and solicits solutions to inform further actions by community organizations and governments.

## Methods

Our research team consists of a female Professor and epidemiologist with extensive global health experience in conducting mixed-methods research studies; a female Associate Professor with a professional interest in addressing healthcare access to reduce health inequities in vulnerable populations; three female research assistants pursuing Master’s degrees in public health related fields with research experience and a passion for community health advocacy; and two community-based organization leaders, one male and one female, who have worked closely with the population being studied. All team members are Asian and committed to ensuring that the research is respectful and responsive to the needs of the community.

We conducted in-depth interviews with Chinese immigrants living in the Greater Boston area to explore their experiences and perspectives on anti-Asian racism during the height of the COVID-19 pandemic. We partnered with the Boston Chinatown Neighborhood Center (BCNC) to recruit participants using a convenience sampling approach and word-of-mouth referrals. BCNC is a longstanding community-based organization (founded in 1969) focused on empowering disadvantaged Asians and new immigrants to build healthy families, achieve greater economic success, and contribute to a thriving community. The majority of clients served by BCNC are Chinese. Information about this study was shared by the research team and BCNC via digital flyers and in hard copy. Bilingual research assistants contacted those who were interested to screen for eligibility. Eligibility for the study included individuals who self-identified as ethnic Chinese; were able to converse in Cantonese, Mandarin, or English; were 18 years of age or older; were knowledgeable about the needs of the local Chinese community; and were able to participate in a one-on-one interview via Zoom. Eligible participants were scheduled for an individual in-depth interview and received the consent script in their preferred language to review on their own before the interview.

Interviews were conducted between June and September 2021 via Zoom or by using the phone feature embedded within Zoom. One research assistant conducted the Mandarin interviews and one research assistant conducted the Cantonese interviews. Both research assistants were trained in qualitative interviewing techniques by the faculty researchers on the project. A verbal consent process was conducted at the beginning of each interview and any questions were answered. For participants who did not agree for their interview to be audio-recorded (*n* = 1), the interviewer took notes. The semi-structured interview guide focused on the impact of Anti-Asian racism on health and well-being during the pandemic. Although the study was conducted during the height of the COVID-19 pandemic, not all of the incidents reported were a direct result of the pandemic. The interview guide also included sociodemographic questions related to age, gender, education, household size, occupation, and years living in the U.S. Each interview lasted approximately 40 min. All participants received a $30 electronic gift card to compensate for their time. This study received ethical approval from Tufts University Social Behavioral & Educational Research Institutional Review Board (ID: STUDY00001487).

### Data analysis

A directed content analysis approach was used to analyze the qualitative data (20). Interviews were analyzed in Chinese (before translation to English) to ensure that the meaning behind codes and key themes would not be lost in translation. Prior to analysis, a list of overarching domains was created to guide the analyses, including effects of the COVID-19 pandemic on individuals, households, and communities; personal experiences of anti-Asian racism; effects of anti-Asian racism; resources for overcoming challenges; barriers to accessing these resources; and recommendations for improving community support. Two research assistants coded each transcript independently and met to discuss and ensure a shared understanding of each code and consistency in its application. All transcriptions and codebooks were subsequently translated into English so that codes and themes could be discussed among the entire study team and issues addressed.

## Results

Twenty-seven participants were recruited from the Chinese immigrant population living in the Greater Boston area. Table 1 shows the sociodemographic characteristics of the participants. Participants ranged in age from 25 to 73 years old and worked in various industries. The majority of participants (92.6%) were female, 70.4% were married, and 66.7% had a high school education or less. The two male participants both worked in the tech industry. Thirteen interviews were conducted in Cantonese, 13 in Mandarin, and one in English.

**Table 1:** Characteristics of interviewees (*n* = 27).

ID	Language	Age	Gender	Education	Marital status	Household Size	Occupation	Years in the U.S.
1	Cantonese	53	Female	< High school	Married	9	Homecare Worker	>10
2	Cantonese	73	Female	< High school	Married	3	Retired	13
3	Cantonese	55	Female	< High school	Married	4	Home Help Aid	21
4	Cantonese	57	Female	Some College	Married	4	Stay home mom	13
5	Cantonese	58	Female	High school	Married	3	Home Help Aid	10
6	Mandarin	27	Female	College	Single	2	Accountant	11
7	Mandarin	25	Female	Master’s degree	Single	3	Accounting Analyst	14
8	Mandarin	49	Female	Associates Degree	Single	2	Personal Care	7
9	Mandarin	54	Female	High school	Divorced	3	Stay home mom	10
10	Cantonese	42	Female	Associates Degree	Married	4	Retail	6
11	Cantonese	35	Female	High school	Married	5	Unemployed	7
12	Cantonese	33	Female	High school	Married	5	Home Help Aid	9
13	Cantonese	37	Female	High school	Married	3	Nursing	15
14	Mandarin	48	Female	< High school	Divorced	3	Certified Nursing Assistant	>8
15	Mandarin	50	Male	College	Single	4	Software Programming	25
16	Mandarin	31	Male	Master’s degree	Separated	2	Software Engineer	9
17	English	46	Female	Master’s degree	Married	4	Stay home mom	Born in U.S.
18	Mandarin	43	Female	High school	Married	3	Assembly line worker	5
19	Mandarin	38	Female	High school	Married	4	Stay home mom	10
20	Mandarin	43	Female	High school	Married	4	Personal care (older person/people)	9
21	Mandarin	35	Female	High school	Married	7	Stay home mom	21
22	Mandarin	34	Female	High School	Married	6	rental assistance coordinator	10
23	Mandarin	33	Female	College	Single	4	Hospital financial analyst	17
24	Cantonese	31	Female	High school	Married	5	Stay home mom	11
25	Cantonese	51	Female	Middle School	Single	2	Helper	20
26	Cantonese	32	Female	High School	Married	4	Stay home mom	9
27	Cantonese	46	Female	High School	NA	4	Stay home mom	13

* NA = Not ask.

### Lived experiences of anti-Asian racism

A majority of participants shared personal incidents of racism and discrimination experienced by themselves, family members, or friends. Table 2 shows the types of discrimination described by community members, with microaggressions being the most frequently discussed type. Research participants often felt they were treated unfairly, stigmatized, or ignored while they were using public services or shopping. In addition, research participants reported several experiences of workplace racism or discrimination. Some participants felt that their opinions were not respected because of their race, while others shared about prejudiced treatment from their supervisors. One participant expressed particular concern over Chinese working in the information technology (IT) industry.

**Table 2:** Anti-Asian racism incidents reported by participants – illustrative quotes by code.*

Microaggressions/Discrimination in various situations	
In accessing public services	“We thought that was discrimination since we have some other friends who had completed the application for the same housing location, and they had not been asked to provide as many materials as this person had been asked for. And those extra materials were hard to obtain so this friend finally lost the housing opportunity.” (ID 10)
	“When I called the services department, they usually called me back very slowly, but when others who were able to speak English made the call, they usually called back much sooner.” (ID 12).
	“When I went to apply or get things, their attitude toward Chinese was not very good. They felt that we did not understand English, so we were not willing to apply for things.” (ID 20)
In stores	“I was …at the cashier line at [retail store]. There was a staff calling people in line to check out at a new open counter. And the Chinese person behind me was trying to remind two non-Asian people [who were standing in front of me] to move to the new line but was verbally attacked by them saying that he [the Chinese guy] had no right to ask them to move.. [one] person looked so aggressive that he looked like he was ready for a fight..” (ID 1)
	“When I was waiting in line for the check-out, one person jumped the queue and stood right in front of me. And he kept staring at me … even as he left the store. I did not dare to look back at him because I was freaked out…” (ID 2)
	“I heard the child ask the father, ‘Why are WE not wearing masks as they do?’ They referred to me and my family…His father said, ‘Because they are Asian’…I was very angry when I heard that at the time.” (ID 7)
	“Recently, when I went to [name of store], the salesperson ignored me and my family for more than 10 or 20 min. She excused herself when I reported her disrespectful behaviors…I feel like we were being treated like air. They ignore our existence. We are treated unfairly, and it seems like we are not welcomed here.” (ID 13)
In the workplace	“My colleague’s son works in the IT industry. He has mentioned the increasing mistrust of the Chinese in this industry…It is basically the conflict between governments, but it is just so unfair to us and our next generation..This will definitely limit their job choices in the future.” (ID 4)
	“…but because he felt he was Asian, if he brought this up, the company would not be willing to listen to his advice” (ID 7)
	“..*[supervisor]* speaking with a tone or a facial expression that is insulting…because it is not once or twice, many times are not ok.” (ID 9)
	“Their [supervisor’s] attitude made me feel like I was causing trouble, and in the end, my attitude was to give up.” (ID 15)
	“I’ve experienced microaggressions throughout my career, throughout school… blatant racism throughout higher-ed, even though my master’s program, microaggressions within family circles at birthday parties for kids, at the school setting from teachers, from other parents that we playdate with.” (ID 17)
Stigmatization	“My family all wore masks and went [shopping]. There was a father and son. That son was very young, he was only eight or nine years old. “I heard the child ask the father, ‘Why are WE not wearing masks like they do?’ They referred to me and my family…His father replied, ‘Because they are Asian’.” (ID 7)
	“At the beginning of the pandemic, even my good friends also asked if this was ‘your Asian virus’ (ID 21)
	“I took the elevator to go downstairs. There was a person in the elevator who saw that I was wearing masks and did not want to be the same elevator with me… The person’s attitude was not asking and I felt discriminated against.” (ID 23)
	“Asians wear masks, people of other races will avoid us when passing by.” (ID 26)

Verbal attacks by strangers	
	“[*My friend’s*] family runs a restaurant.. many unknown people called to his restaurant to curse him, because everybody knows that it is a Chinese restaurant. They called to scold my friend…” (ID 7)
	“Once I was walking on the road, there was a person who said “Chin-English,” but he passed by quickly” (ID 8)
	“Once in Boston, there was a homeless person on the street. It was relatively late. When there was no one on the road, he would ask for money when you pass by. I said, ‘oh sorry, I do not have one’, he said something such as, ‘go back to your country’, or other words.” (ID 16)
	“…as early as March 2020 I heard friends getting verbally assaulted in NY City on public subway, and then in different parts of the tri-state.” (ID 17)
	“Someone spit on me and shouted, ‘shut the [expletive] up, your Asian virus go away’” (ID 21)
	“During the lockdown I was scolded by others for no reason in the supermarket parking lot.” (ID 22)

Violent attacks on older person/people Asians	
	“…something that happened to my granddaughter’s classmates’ grandma. She was robbed by a [man] when she came out from [retail store]. She did not allow the robber to take her bag so she was beaten by the robber and she needed to go to the hospital. I have seen her photos that her face was badly hurt (swollen and bruised). It makes me so scared to go out at night, not even after 6 pm..” (ID 2)
	“The grandmother of my daughter’s friend got hit a few days ago, around May 20th. Her face was seriously hurt and she needed to go to the hospital. It was said that the grandma was hit on her way back home from a walk, and her face was severely hurt. But she was attacked randomly, you know.” (ID 3)
	*[Referring to a previous incident where an aunt was beaten] “*It was so panic and painful in my heart. After all, a person who is such a close family member.. I feel that such a person who harms the older person/people for no reason is really inhumane and excessive.” (ID 14)

* A participant can report multiple types of racism experienced.

Many participants felt strongly that Asians or Chinese were stigmatized due to COVID-19 and mask-wearing. Blaming the Chinese for the pandemic was linked to *“leaders’ remarks”* (ID 22) and *“misconceptions led by the media”* (ID 4). One participant noted that new COVID-19 variants had been found in other countries, but the Chinese were the ones that were stigmatized. Some participants also reflected on how they were avoided or discriminated against by others when wearing masks.

Another common type of discrimination shared by participants was verbal assaults. Research participants reported insults thrown at them when merely walking down the street. One participant further shared how she worried about her friend, a Chinese restaurant worker, who was constantly being harassed by unknown people calling the restaurant during the pandemic. In addition, two participants described violent attacks on older person/people Asians who were severely harmed and hospitalized. One of them shared that the grandmother of her daughter’s friend *“was hit on her way back home from a walk, and her face was severely hurt.”* (ID 3).

Our participants reported on a wide range of discriminatory incidents, both experienced or observed, but we did not note any associations between participants’ characteristics and the type of discriminatory experiences they reported.

### Isolation

While social distancing and quarantine reduced everyone’s connection at the beginning of the COVID-19 pandemic, members of the Chinese community appeared to experience more extreme isolation over a longer period. Some participants expressed that the Chinese community, in general, tends to be more cautious about COVID-19 compared to others and are therefore more inclined to reduce contact with other people, even when restrictions were lifted. In addition, several participants shared that they were especially scared to go out given their awareness of specific anti-Asian racism incidents taking place in the community and across the country. *“Trapped at home”* was the term used by one participant (ID 12) to describe the situation. Several participants mentioned that they specifically directed the older person/people in their households to not take public transportation, go grocery shopping, or even walk outside.

*“I think I've never before felt scared for my parents the way I do now, especially because the older person/people were being attacked so blatantly and they still are. So, I've never had to have the conversation with my parents, like don't go outside [until now].” (ID 17)*

### Coping strategies

More than half the participants reported modifying their own behaviors to adapt to the challenging environment. Different types of strategies were described including isolation, avoidance, staying alert, and preparing themselves with defensive tools or weapons.

Many participants chose to avoid going to public spaces where incidents were likely to occur.

Others avoided going out at night and were extra careful when taking the subway, staying away from the edge of platforms. For safety reasons, younger adults also reported changing their daily routines and becoming more alert when outdoors.

*“I will avoid areas … like a subway station or a place where many homeless people gather. It was unsafe already and could be even more dangerous during these turbulent times. It feels like violent people or people with bad behaviors may be more likely to be in those places.” (ID 7)*

Some participants expressed the importance of being advocates and speaking up for themselves and others against discrimination.

*“I just think it is my responsibility … I hope more people will understand. If I can do something and you can do a little bit, and we tried to stop it from getting worse, then why not?” (ID 7)*

However, most of our participants were hesitant or afraid to speak up, primarily due to concerns about personal safety. For example, one participant expressed:

*“I am afraid that if I argue with the person, it will escalate the issue into a physical conflict and threaten my physical safety.” (ID 16).*

Many weighed the potential costs and benefits of fighting back when encountering racist events, but in most cases, they felt the costs outweighed the benefits and that prevented them from defending themselves. Participants felt that they were in a helpless situation and believed that keeping silent was the best option for them. As one participant reflected:

*“Many Chinese do not know what to do when they encounter these problems. They do not speak English and it is difficult to ask for help. Moreover, many Chinese are unwilling to help out when they see this kind of thing.” (ID 27)*

Consequently, most parents of school-age children instructed their children to *“not have conflicts”* (ID 26) and *“stay away from people”* (ID 25) in order to avoid problems.

When discussing incidents of workplace discrimination, participants mentioned some actions that were taken by the victims against unfair treatment. One reported success after getting support from the workplace.

*“… then she [participant’s daughter] went to [her boss], and then her [workplace] had a multi-ethnic and multi-racial meeting, and then finally she felt that she won because the boss was transferred.” (ID 9)*

Other participants, however, were not as successful:

*“… the [supervisor’s] attitude made me feel like I was causing trouble, and in the end, my attitude was to give up.” (ID 15)*

The feeling of living in *“someone else’s country”* (ID 15) further constrained this participant from asking for equality and respect.

Some emphasized the importance of learning self-defense and being physically prepared for attacks. Tools like security cameras, pepper spray, and other weapons provided them with a sense of security to be able to defend themselves in case of an attack.

### Health effects

Health and safety concerns significantly impacted the mental and emotional well-being of community members. Worry, stress, fear, anger, depression, and sleep disturbances were commonly reported by community members. Participants felt strongly that anti-Asian racism experiences continued to have negative effects on mental health.

Participants expressed feeling very conflicted because although they were angry at being targeted, they were also worried about escalating the situation. Several participants discussed how they did not feel secure and were constantly worried about being hurt by people around them.

*“You worry about going out, you must be more careful, you are going to be paranoid, and you need to be highly vigilant. You think about whether the person around you may hurt you. In the long term, you are overly nervous.” (ID 8)*

Unstable mood was another manifestation resulting from worry – not only about their own circumstances but also about the safety and isolation of their older person/people parents.

*“Because I live in a senior house and take care of my father, I can especially feel that the quality of life of the older person/people has changed a lot. They stay at home and are afraid of going out. They are very nervous, so as their offspring … I am a lot more irritable than before, and my mood is not very stable, because you don’t go to work, you have less money, there are a lot of messes, and I have to worry about what happens to my father.” (ID 9)*

In addition to their own mental health, some spoke of the negative effects of racism on the younger generation and the views that children were developing about themselves and the world. One participant stated,

*“… any toxic environment will affect the victims’ mental health. If this phenomenon is very common, then many Asian children will be traumatized. Because discrimination, no matter what form, time, or context, will have a negative impact on people's mental health.” (ID 6)*

Although most parents stated that their children had not encountered anti-Asian racism, they still worried about how their children might be bullied or attacked at school. Parents believed that *“children are impacted by their parents’ ideas”* (ID 21). The influence that parents have on their children’s attitudes is highlighted by the story shared previously from the participant who overheard a non-Asian father explaining to his child in the store aisle that the reason the participant’s family was masked was *“because they are Asian”* (Table 2, Stigmatization, ID 7).

### Resources and barriers to accessing resources

Community members pointed out some of the existing resources that they found helpful to combat the rising crisis of anti-Asian racism. Support from the workplace was mentioned most frequently. For some, their workplace offered support by having regular and open discussions about racial justice or providing individual support through HR departments. One participant shared her experience at the workplace:

*“In my company, there is an HR department. There are no support groups dedicated to this, but if you have anything [race-sensitive experiences], you can go to them.” (ID 6)*

A few parents commented about the schools’ approach to racial justice. Some schools held meetings for faculty on a regular basis to discuss racial equity issues. Many parents trusted the ability of teachers and schools to handle conflicts and incidents. Family liaisons at schools and in the Department of Education were mentioned several times by parents as a resource.

Participants mentioned several types of social media and other platforms used to disseminate information to the community. “Stop Asian Hate”-related YouTube campaigns, WeChat petitions, online and in-person community meetings, flyers with safety information, and “Stop Asian Hate” signs in the community all contributed to increasing awareness of racial injustice in the Asian community. Participants were also aware of several local organizations in Chinatown and the Greater Boston area that were dedicated to supporting Asians.

Although resources exist and are helpful in supporting the community, not all participants were able to easily access them. Some of the major barriers preventing people from getting help included a lack of knowledge about existing resources, language barriers, financial constraints, and limitations of the existing programs. Some participants indicated that they were either not aware of community resources or did not know how to access the resources they heard about. One participant shared,

*“I think such an organization definitely exists [for mental health counseling], but I don’t actually know how to access it. So, I think it would be very helpful if someone can find this resource very simply when they have needs.” (ID 6)*

Individuals with limited English proficiency found it especially challenging to request help, and it often took extra effort and money to access simple social services. One participant provided an example about contacting the police during emergencies,

*“The older person/people know the phone number to dial [911] but they are not able to communicate with police when they encounter problems.” (ID 14)*

Language and cultural barriers also limit the services available to immigrants. Participants commented that they had to rely on organizations that speak their language. One participant was struggling to find a culturally competent therapist who would understand her perspective. Some expressed such difficulties in overcoming these barriers that they lost trust in their community’s ability to support their needs. One participant expressed disappointment when asked about which community resources they accessed – *“Nothing. I can only do my part by myself”* (ID 24).

### Proposed solutions

Community members were asked to provide suggestions on how to fight against anti-Asian racism. Five main suggestions were identified from the responses: (1) improve education on racial equity, (2) enhance support within the AAPI community, (3) encourage social advocacy to make AAPI voices heard, (4) create alliances with other BIPOC communities, and (5) improve government support and protection for the community.

(1) Improve education on racial equity.

Participants mentioned that they hoped to see more education on racial justice. Increasing awareness of racial justice comprehensively, regardless of age and background, would be a fundamental strategy for improving racial justice. As one participant stated, *“it begins with education and there is not an age that’s too young”* (ID 17). Another participant suggested, *“the school can provide a brochure telling children what to do* [when they are bullied]*”* (ID 28).

(2) Enhance support within the AAPI community.

Although participants felt it was important for Asians to shift from avoidance to advocacy, the importance of having empathy and support was also recognized. Establishing community support groups and providing safe spaces for people to communicate thoughts, share experiences, and discuss difficulties was suggested as a potential way to provide direct help to community members. One participant suggested:

*“… just provide them [Chinese community] with such a forum … a safe place to express their unhappiness or whatever they encountered, that will be good”. (ID 9)*

Another suggestion was for community organizations to provide counseling services targeted at racism-related issues. Specifically, one participant commented:

*“It would be good for organizations to hold meetings for educational purposes about equal rights. And also, they [organizations] can help the victims who suffered from racist incidents. They will provide us with emotional support as a group”. (ID 2)*

(3) Encourage social advocacy to make AAPI voices heard.

Participants shared similar opinions about the importance of speaking up and raising awareness within and outside the Asian community. Participants felt that not everyone may be aware of the severity of the racial injustice facing the Asian community, so people should speak out about their racist experiences in community forums and through awareness campaigns. One participant used the Black Lives Matter and the LGBTQ movements as examples of successful campaigns that helped to draw society’s attention.

*“Like LGBTQ groups, and then the black community, they have been successful in getting their voice out, and the public has heard it. For example, June is now Pride Month. So I think that LGBTQ groups can stand up for themselves, and at the same time, they can show their true faces to everyone, not be demonized or treated with prejudice, and then let everyone correctly understand who they are as groups. So this is a successful campaign that I think is relatively healthy.” (ID 6)*

Publicizing the issue through newspapers, on TV, and in social justice campaigns was also recommended. However, as mentioned previously, the lack of a sense of security was a major concern preventing people from speaking up individually about racial encounters. In many cases, people were aware of the injustices and wanted to make changes, but perceived threats prevented them from speaking up. Cultural barriers were also mentioned as a reason why people did not speak up.

*“There's a whole Saving Face thing, like you know a lot of people within our culture just aren't going to broadcast it. Even if it's dire and terrible and people should know for their own safety, I think that's just like an ingrained part of our overall Asianness – to keep quiet and low-key.” (ID 17)*

(4) Create alliances with other BIPOC communities.

Building upon social advocacy, community members hope to improve cross-ethnic collaboration and communication to reduce misunderstanding and increase appreciation of different cultures and perspectives. As one participant believed, *“It’s really powerful when there are alliances and community between each other, whether it’s across ethnicities or across gender … Groups need to talk”* (ID 17) Such connections can also foster empathy and trust, as well as make individuals and communities feel more included. One participant shared that *“a lot of friends around me were not Asian, but when they heard about my experience or the experience of my Asian friends around me, they were willing to offer help”* (ID 7).

(5) Improve government support and protection for the community.

Furthermore, participants spoke about their wish for more government-level effort to be devoted to helping address the difficulties faced by Asians. One community member hoped that *“the government will make a correct statement, not to say that this is a Chinese virus”* (ID 21).

Specifically, participants wanted to see increased police presence and response to enhance their sense of security in the community, such as having more police patrols. One participant suggested that:

*“for places where these things [physical attacks] frequently happen, patrol cars should move around more often. We must increase efficiency and adopt such measures”. (ID 14)*

Another participant shared:

*“For my robbery experience, I waited for more than twenty minutes to have the police arrive to help me although the police station was only a 5-minute walk to the place where I was robbed.” (ID 1)*

Improving legislation on hate crimes to support minorities with legal protection was also strongly desired. More than one participant pointed out the importance of having strong leadership that represents the voice of minorities. Participants hoped that:

*“… in the future, there will be more Asians or people of Chinese descent who can enter politics or reach a higher level in society, a higher position. And if they can make use of their social resources, they will be influential. People will respect them, listen to them, and then they can make our voices heard.” (ID 16)*

## Discussion

The HEAR US study reveals multiple effects of anti-Asian racism on a community of Chinese immigrants during the COVID-19 pandemic. Although we do not have any historical comparisons, our data captured the heightened fear and anxiety of attacks that participants associated with the amplification of anti-Asian hate crimes during that period. Compared to previous studies on this topic which were primarily conducted using quantitative surveys, our qualitative results provide more vivid and nuanced descriptions of the lived experiences of this community, depicting the overt and inner struggles of the community and its effects on health. Our qualitative interviews went further to solicit ideas and solutions from participants that would be helpful to the community, information that previous quantitative surveys did not provide.

For the most part, the major themes obtained from our interviews agree with results from previous quantitative surveys and are not unique to the Greater Boston area. Our findings suggest that anti-Asian racism was widely experienced by community members, with microaggression being the most reported type of incident followed by verbal assaults and physical attacks. The extensiveness of racist incidents that we discovered locally resonate with the findings from surveys that explored the discrimination experience of the AAPI population nationally (10, 12, 14). For example, the COMPASS Study, a nationwide community-based survey, showed that 60.7% of its participants reported discriminatory experiences, and the NAPAWF revealed that 74% of the AAPI women who participated reported such experiences (10, 14). For the types of racism incidents, our findings align with another interview-based study that explored the discrimination experience among Asian healthcare workers, which highlighted the common occurrence of microaggressions against Asian healthcare workers in both the hospital setting and outside environment during the height of the COVID-19 pandemic (16). Our study also finds that verbal assaults and physical attacks are two other types of incidents that are commonly reported by the research participants.

Although many participants felt that Boston was safer compared to other areas, such as New York and California, all 27 participants were easily able to share incidents of perceived racism experienced personally or by friends and family members (14). It was noted in a prior national survey that hate crimes are usually underreported since people often lack the literacy, resources, or simply courage to report such events (13). Although the racism incidents reported in our study are mainly microaggression and verbal assaults, and not physical attacks, reports of violence against Asians elsewhere can still have significant effects on individuals, especially on their sense of security.

The health effects reported by our participants encompass emotional, mental, and physical health effects. Our results align with findings from both regionally and nationally representative surveys and demonstrate the negative association between discrimination and well-being (8, 21, 22). A cross-sectional study that surveyed Chinese and South Asian adults in Chicago found that the prevalence of depressive symptoms among these groups had doubled since the pandemic began (23). Other survey-based studies found that high proportions of participants reported feeling stressed, anxious, depressed, or distressed about discriminatory experiences (10, 12, 15). Among our participants, fear, anxiety, stress, helplessness, a sense of insecurity, and anger are all mentioned as emotional and mental health effects resulting from experiences of racism and discrimination. These negative psychological effects are similar to findings from qualitative studies conducted in other populations of Asians. The qualitative study by Shang et al. in Asian healthcare workers in Canada and the US reported that experiences of threats of violence or actual physical assault linked to COVID-19 resulted in a range of negative emotions and cognitive processes, such as despair, rumination, and hypervigilance among the victims (16). In a study of Asian international students in the U.S., participants reported similar mental health challenges facing this ethnic group during COVID-19, mainly attributed to the rise of anti-Asian racism, the lack of sense of belonging and social support, and the uncertainties around immigration status related to the unexpected lockdown (19).

Chronic mental stress can lead to changes in physical health, as reported by our participants (24, 25). Sleep disturbance and deterioration of sleep quality are the major physical manifestations of the stress experienced by participants during this period. A similar observation was made in a prior online survey among Asian American adults as well as in a longitudinal study among Asian American adolescents, demonstrating the association between racism-related experiences and poor sleep quality (21, 26).

Many of the health effects mentioned by the participants, such as anxiety and sleep disturbances are symptoms of depression. However, few of the participants specifically used the Chinese word for “depressed” or “depression” in their descriptions. For cultural reasons, many Asian immigrants are hesitant to link their experiences to psychological changes and to seek mental health services (15, 18, 27). They are generally afraid to bring stigma and shame upon themselves and their families due to the perceived discrimination associated with mental health conditions (28). In other words, although mental health impacts were mentioned in interviews, it may be just the tip of the iceberg. Culturally, people of Asian descent may unconsciously downplay or ignore the impact of the challenging events on their health and not realize the long-term consequences. This issue is further compounded by the lack of culturally competent mental health providers, as shown in the literature (27, 29, 30). Improving access to culturally and linguistically appropriate mental health services is urgently needed in this population.

When asked about the resources that Asian immigrants can access to address challenges and the barriers they faced when seeking help, participants indicated that they were either not aware of community resources or did not know how to access the resources they heard about. Only a few community organizations were named as being helpful in addressing the issue of anti-Asian racism.

When asked for suggestions on how services can be improved, participants mentioned building social support as one of the key strategies. Participants suggested that local communities set up support groups to develop interpersonal connections, enhance racial identity and pride, and provide guidance for people in need. They noted that community organizations can help improve access to existing resources and provide a forum to build emotional support and courage to speak up for their rights. Where possible, to build collective efficacy, organizations should hold conversations about how to implement emotional support services more effectively, ensuring such services are easily accessible to community members and safe spaces are available for discussion around sensitive topics like racism. Teaching community members to advocate for themselves to speak up against racism may be necessary to address cultural norms of staying silent and avoiding conflicts. Recognizing the limited funding and capacity of many organizations, it is important to have cross-sector learning and to invest in local community-based organizations so that they can provide linguistically and culturally responsive services. Community members also expressed interest in building alliances across ethnic groups to foster inter-racial communication and understanding to strengthen the respect between racial groups. A nationally representative survey conducted by the NAPAWF also emphasized the role of community partnerships as being the crucial bridge between individuals and societal resources (10). Finally, education on racial equity in school-age children is another important strategy mentioned by participants which will help to address the issue from a young age. Family-oriented interventions to teach both parents and kids how, when, and to whom incidents of bullying and discrimination should be reported can also help foster communication about racial injustice between parents and their school-age children.

From the systems level, developing culturally and linguistically appropriate resources and tools will be necessary to support Asian immigrants’ health, and such a strategy is also applicable to assist other minority groups. More governmental level support is desired as well, to ensure basic social security and fundamental justice against discriminatory events. Although federal-level hate-crime legislation was enacted in 2021, more support from local governments is crucial to maximize the effect of the federal law (31).

Our study has several strengths. The qualitative nature of this study allows participants to share their stories more deeply and reflect on their culture and identity. The use of the interpretive paradigm in this study is another strength, which helps value the social contexts when understanding human behaviors (32). In addition, our study is unique in that bilingual interviewers helped to facilitate trust building with participants, which allowed for deeper narratives as participants could more easily express their thoughts and opinions in their native language.

Although our study provides us with an in-depth understanding of the effects anti-Asian racism, there were some limitations to our study. Firstly, due to the qualitative nature of this study, we are not able to quantify the prevalence and severity of racist events, which could be helpful in determining the intensity of anti-Asian racism specifically in the Greater Boston area. Nevertheless, the primary purpose of our study was not to measure the extent of anti-Asian discrimination, but rather to offer context and amplify the voices of those affected. By exploring their thoughts, emotions, and reactions to these incidents and proposing solutions from the community, our findings still have significant implications for society.

Secondly, since this study is cross-sectional in design and cannot establish a causal relationship or indicate whether discrimination increased during the pandemic. We did not ask these questions directly in our interview and no pre-pandemic studies are available for comparison. Although we can’t directly attribute discrimination and racial hate to the pandemic, it is important to acknowledge that the COVID-19 outbreak acted as the major catalyst for the collective awakening and reporting of anti-Asian racism across the country, making this issue worthy of investigation.

Thirdly, participants were purposefully recruited to share their observations and personal struggles with anti-Asian racism, which could introduce some bias in the stories and opinions expressed during the study. Being aware of this potential bias can help contextualize and interpret the narratives provided. For example, the majority of participants were female, so male perspectives were lacking. Also, our study included only Chinese immigrants, which limits our understanding of what other Asian ethnic groups experienced during the pandemic. However, within these subgroups, we were able to obtain perspectives from a diverse group with respect to language (Cantonese vs. Mandarin), age, education, marital status, and occupation.

The HEAR US study showcases the unique struggles the Chinese community faced during the COVID-19 pandemic amid the rise of anti-Asian racism in the U.S. and reveals just how common experiences of racism and discrimination are in people’s daily life. To effectively restore and better support the well-being of this minority group, future actions can be taken such as improving education on racial justice; improving self-efficacy to speak out against racism; encouraging social advocacy; reinforcing collective discussion between groups; enhancing community and government support of culturally and linguistically appropriate services; and having representative leaders that speak in the voice of the community. The HEAR US team will continue to work with local community-based organizations to develop resources and programs to provide targeted support. As society becomes more diverse and connected, more studies to understand the challenges faced by immigrants will be helpful to promote health equity and healthy lives.

## Data availability statement

The raw data supporting the conclusions of this article will be made available by the authors, without undue reservation.

## Ethics statement

The studies involving humans were approved by Tufts University Social Behavioral and Educational Research Institutional Review Board (ID: STUDY00001487). The studies were conducted in accordance with the local legislation and institutional requirements. The ethics committee/institutional review board waived the requirement of written informed consent for participation from the participants or the participants’ legal guardians/next of kin because the interviews were conducted remotely during COVID-19, and the participants provided verbal consent.

## Author contributions

The authors confirm that AT, KD, YY, and BH contributed to the conception and design of the study. SC, YL, and SL were involved in data collection and analysis. All authors discussed and contributed to interpretation of results. SC and YL drafted the manuscript. All authors contributed to the manuscript revision, read, and approved the submitted version.

## Funding

This work was supported by the Tufts University Springboard funding mechanism from the Office of the Vice Provost at Tufts University.

## Conflict of interest

The authors declare that the research was conducted in the absence of any commercial or financial relationships that could be construed as a potential conflict of interest.

## Publisher’s note

All claims expressed in this article are solely those of the authors and do not necessarily represent those of their affiliated organizations, or those of the publisher, the editors and the reviewers. Any product that may be evaluated in this article, or claim that may be made by its manufacturer, is not guaranteed or endorsed by the publisher.
